# A novel CT-based radiomics model for predicting response and prognosis of chemoradiotherapy in esophageal squamous cell carcinoma

**DOI:** 10.1038/s41598-024-52418-4

**Published:** 2024-01-23

**Authors:** Akinari Kasai, Jinsei Miyoshi, Yasushi Sato, Koichi Okamoto, Hiroshi Miyamoto, Takashi Kawanaka, Chisato Tonoiso, Masafumi Harada, Masakazu Goto, Takahiro Yoshida, Akihiro Haga, Tetsuji Takayama

**Affiliations:** 1 Department of Gastroenterology and Oncology, Tokushima University Graduate School of Biomedical Sciences, 3-18-15 Kuramoto-cho, Tokushima, 770-8503 Japan; 2 Department of Gastroenterology, Kawashima Hospital, 6-1 Kitasakoichiban-cho, Tokushima, 770-0011 Japan; 3 Department of Radiology, Tokushima University Graduate School of Biomedical Sciences, 3-18-15 Kuramoto-cho, Tokushima, 770-8503 Japan; 4 Department of Thoracic, Endocrine Surgery and Oncology, Tokushima University Graduate School of Biomedical Sciences, 3-18-15 Kuramoto-cho, Tokushima, 770-8503 Japan; 5 Yoshida Clinic, 1-18 shinuchimachi, Tokushima, 770-0845 Japan; 6 Department of Medical Image Informatics, Tokushima University Graduate School of Biomedical Sciences, 3-18-15 Kuramoto-cho, Tokushima, 770-8503 Japan

**Keywords:** Predictive markers, Oesophageal cancer, Oesophageal cancer, Tumour biomarkers, Chemotherapy, Radiotherapy

## Abstract

No clinically relevant biomarker has been identified for predicting the response of esophageal squamous cell carcinoma (ESCC) to chemoradiotherapy (CRT). Herein, we established a CT-based radiomics model with artificial intelligence (AI) to predict the response and prognosis of CRT in ESCC. A total of 44 ESCC patients (stage I-IV) were enrolled in this study; training (n = 27) and validation (n = 17) cohorts. First, we extracted a total of 476 radiomics features from three-dimensional CT images of cancer lesions in training cohort, selected 110 features associated with the CRT response by ROC analysis (AUC ≥ 0.7) and identified 12 independent features, excluding correlated features by Pearson’s correlation analysis (r ≥ 0.7). Based on the 12 features, we constructed 5 prediction models of different machine learning algorithms (Random Forest (RF), Ridge Regression, Naive Bayes, Support Vector Machine, and Artificial Neural Network models). Among those, the RF model showed the highest AUC in the training cohort (0.99 [95%CI 0.86–1.00]) as well as in the validation cohort (0.92 [95%CI 0.71–0.99]) to predict the CRT response. Additionally, Kaplan-Meyer analysis of the validation cohort and all the patient data showed significantly longer progression-free and overall survival in the high-prediction score group compared with the low-prediction score group in the RF model. Univariate and multivariate analyses revealed that the radiomics prediction score and lymph node metastasis were independent prognostic biomarkers for CRT of ESCC. In conclusion, we have developed a CT-based radiomics model using AI, which may have the potential to predict the CRT response as well as the prognosis for ESCC patients with non-invasiveness and cost-effectiveness.

## Introduction

Esophageal cancer (EC) is the eighth most common malignancy and the sixth leading cause of cancer-related death worldwide^1^. Esophageal squamous cell carcinoma (ESCC) accounts for almost 80% of all EC cases and ESCC is one of the deadliest cancers due to its highly aggressive nature and poor survival rate^2^. Chemoradiotherapy (CRT) is one of the most effective treatments for ESCC because CRT can potentially be curative and is less invasive than surgery; in contrast, esophagectomy remains highly invasive and is sometimes correlated with postoperative morbidity and mortality^3–5^. Moreover, CRT is widely applicable to early-stage and locally-advanced ESCC, even at the palliative stage. Despite the effectiveness of CRT for ESCC, a certain population of patients who undergo CRT experience subsequent recurrence within a relatively short period^6^. The resistance to CRT is one of the major causes of treatment failure in patients with ESCC^7^. However, the molecular characterization of CRT resistance is very complex, and it is extremely challenging to identify and decode the mechanism of CRT resistance using a basic biological approach. Therefore, it is necessary to find optimal clinical biomarkers that can distinguish responding and non-responding patients with ESCC.

Radiomics is a new quantitative analysis approach to medical imaging. The information generated about a large number of image features within tumors including their spatial and temporal heterogeneity can be applied to create diagnostic, prognostic, and predictive models. Radiomics analysis can be performed by extracting quantitative radiomics features from multimodality medical images, such as ultrasound (US), computed tomography (CT), magnetic resonance (MR), and positron emission tomography (PET) scans^8–12^. Recently, the technology of CT analysis has enabled high-level quantitative evaluation of features and pixel-based textures for tumor characterization^13^. Furthermore, machine-learning algorithms of artificial intelligence (AI) using CT images are boosting the powers of radiomics to predict treatment response and prognoses^14^. Such recent advances in radiomics technologies have opened a new era of radiomics-based biomarker discovery, which can reveal in-depth tumor characterization. In addition, the availability of large amounts of medical data, together with advanced computerized image analysis approaches with AI have paved a new path for identification of more precise and robust biomarkers.

Therefore, in this study, using a systematic and comprehensive biomarker discovery process with AI, we compared CT-based radiomics features of ESCC between responder and non-responders, established a novel, non-invasive, radiomics prediction model and then validated the model in an additional cohort. Moreover, using Kaplan–Meier survival analysis, we evaluated the model’s performance in predicting the prognosis of CRT in ESCC patients. Finally, we performed univariate and multivariate Cox regression analysis to show superiority of the AI based radiomics model.

## Methods

### Patients and study design

This was a retrospective single-institution study at Tokushima University Hospital (Tokushima, Japan). We continuously enrolled a total of 50 patients with pathologically proven ESCC who underwent CRT as first-line treatment from February 2009 to September 2019, and generated datasets on February 24, 2022. Of these patients, 6 were excluded from this study due to lack of clinical information such as accurate survival time, and the remaining 44 were analyzed. Among the 44 patients, 27 were admitted and received CRT in the Department of Gastroenterology and Oncology, and 17 were in the Department of Thoracic, Endocrine Surgery and Oncology of Tokushima University Hospital. Because the ratio of the training cohort and validation cohort is reported to be approximately 6:4^15^, we used the former as a training cohort and the latter as a validation cohort.

**Figure 1:**
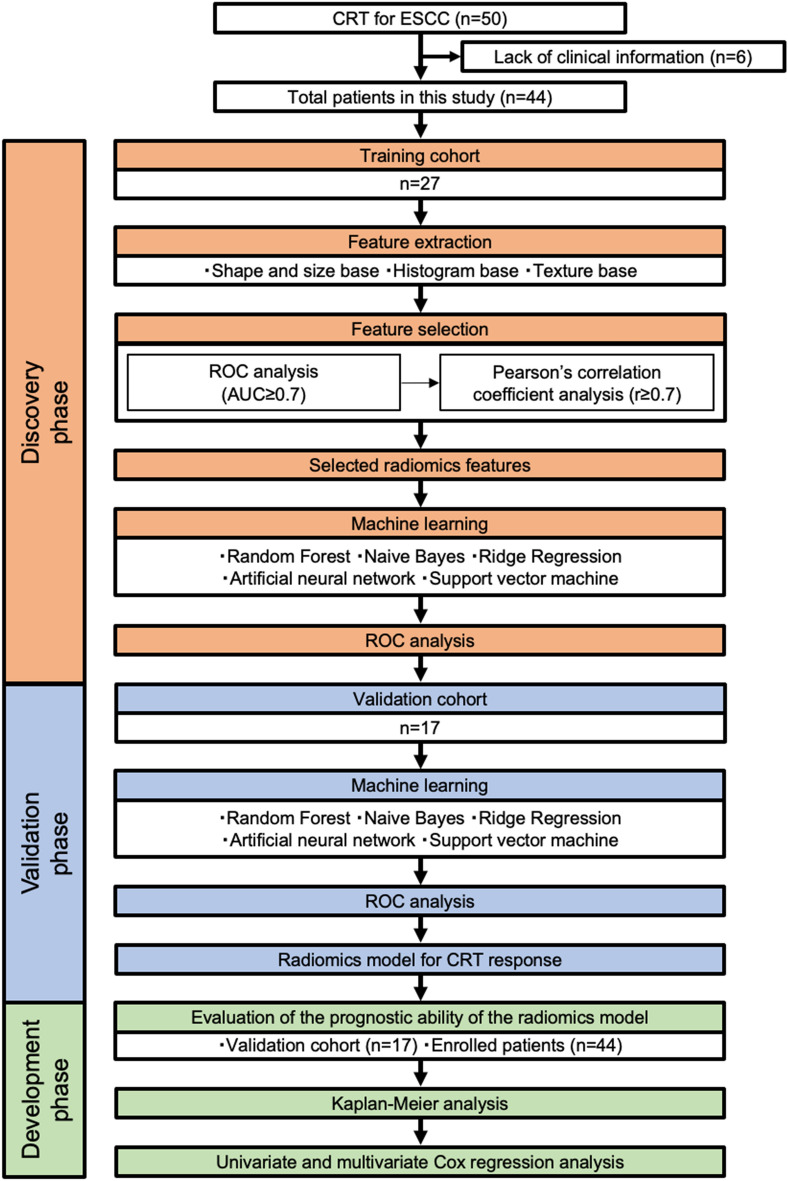
Study design for the identification and validation of the CT-based radiomics model for predicting response to and survival following CRT in ESCC. Among 50 patients with esophageal squamous cell carcinoma (ESCC) who underwent CRT, we excluded 6 patients from this study due to lack of clinical information such as accurate survival time. Ultimately, 44 patients were enrolled. Radiomics features were extracted and selected from the CT images for each patient. We created 5 machine learning models using radiomics features from the training cohort (n = 27) and selected the best model using ROC analysis. We evaluated the best-performing prediction model using a validation cohort (n = 17). Survival analysis was performed using the validation cohort (n = 17) and all cases (n = 44).

To identify a novel CT-based radiomics model associated with the CRT response in ESCC patients, we designed this study in 3 phases: a discovery phase for the selection of candidate radiomics features and construction of the prediction model, a validation phase with an independent CRT clinical validation cohort to assess the performance of the radiomics prediction score as a CRT response marker, and a development phase with the validation cohort (n = 17) and all enrolled CRT patients (n = 44) to assess and advance our CRT response marker as a prognosis-prediction marker as well (Fig. 1).

Cancer staging was performed according to the Union for International Cancer Control TNM staging system (8th Edition). All patients underwent esophageal endoscopy for endoscopic and histological evaluation of the effect of CRT 1 month after completion of CRT. The median follow-up time of all the patients analyzed was 63.4 months (95%CI 46.5–80.2). This study was conducted in accordance with the Declaration of Helsinki and approved by the ethics committee of Tokushima University Hospital. Informed consent was obtained from all patients prior to the collection of any data.

### CT imaging protocol

All patients were examined using a 16-detector row Aquilion LB model CT scanner (Toshiba, Tokyo, Japan). The CT scanning parameters included a tube voltage of 120 kV, tube current auto, pixel size 0.976 × 0.976 mm^2^, and slice thickness 2.5 mm. All raw data were reconstructed with a 0.625 mm section thickness for the routine axial CT images. No patient received intravenous contrast medium.

### Chemoradiotherapy

During the whole course of radiotherapy, patients underwent 2 cycles of chemotherapy. The chemotherapy regimens used were as follows: NF (nedaplatin 70 mg/m^2^ + 5-fuluorouracil (5-FU) 700 mg/m^2^) for 16 cases, FP (cisplatin 60 mg/m^2^ + 5-FU 600 mg/m^2^) for 8 cases, DNF (docetaxel 30 mg/m^2^ + nedaplatin 50 mg/m^2^ + 5-FU 400 mg/m^2^) for 8 cases, and DFP (docetaxel 25 mg/m^2^ (weekly) + cisplatin 6 mg/m^2^ (day1-5) + 5-FU 370 mg/m^2^) for 12 cases. All patients received 50.4 Gy/28 Fr (n = 27) or 60 Gy/30 Fr (n = 17).

### Treatment evaluation

A responder to CRT was defined as `complete response (CR) of primary lesion in the radiation field’ maintained for more than 1 year. Evaluation of the CRT response was performed 1 month after the completion of CRT by CT scan and endoscopy. CT scans were then taken every 3–6 month for 2 years, and approximately every 6 months since then in all patients. According to the criteria from 11th edition of the Esophageal Cancer Handling Regulations, endoscopic primary lesions were evaluated as follows: (1) all endoscopic findings suggestive of neoplastic lesions have disappeared, (2) there is pathologically no cancer detected by endoscopic biopsy of the primary lesion that was present before treatment, (3) the entire esophagus can be observed by endoscopy, (4) there are no endoscopic findings suggestive of active esophagitis (no swelling alteration, no white moss). A complete response was achieved when all of the above findings (1) to (4) were satisfied^16^. Patients who did not meet the response definition were categorized as non-responders.

### Feature extraction

We extracted radiomics features from each pretreatment CT images. A schematic illustration of the process of extracting radiomics features is shown in Supplementary Fig. S1. First, the volume of interest (VOI), which is equivalent to gross tumor volume (GTV) in the treatment planning of radiotherapy, was manually delineated by the same radiologist (T.K.) to mitigate intra-observer delineation variability. The VOI was set on the three-dimensional (3D) CT image for all patients, and then 8 features depending only on the shape and size of the VOIs were extracted. A 3D wavelet transform was applied to each CT dataset to decompose into 8 components for extraction of the histogram and texture features. All decomposed images as well as the original image were resampled isotropically with a 2-mm scale and were requantized with a 25-Hounsfield unit bin size. Then, 10 × 9 (90) histogram features were extracted from each component image as well as from the original image. Similarly, 42 × 9 (378) texture features were extracted. Thus, 1 case has 476 features extracted from original and wavelet filtered images. Through the feature extraction process, we modified MATLAB programming tools for radiomics feature extraction^17,18^.

### Feature selection

In the discovery phase, we selected candidate radiomics features from the CT images, which associated with CRT response in the training cohort. We calculated AUC value for each of all features for response by receiver operating characteristic (ROC) analysis using c-statistics, and selected radiomics features which significantly associated with responders (AUC ≥ 0.7, *p* < 0.05). Furthermore, to avoid redundancy for such selected features, we used Pearson’s correlation coefficient analysis and limited the feature spaces by discarding features that were highly correlated with the others. In this study, we used r ≥ 0.7 (*p* < 0.05) as the threshold value for the pairwise correlation^19–21^.

### Machine learning

We used 5 commonly machine learning algorithms to achieve the best predictive model. These machine learning algorithms including Random Forest (RF) model, Naive Bayes (NB) model, Ridge Regression (RR) model, Artificial Neural Network (ANN) model, and Support Vector Machine (SVM) model were compared based on ROC curves and the best-performing prediction model was selected^22–24^. In the validation phase, we evaluated the models constructed in the discovery phase to discriminate between responders and non-responders by ROC analysis for the validation cohort.

### Prognosis analysis

In the development phase, to evaluate whether our radiomics model is able to predict prognosis as well, Kaplan–Meier analysis was performed comparing progression-free survival (PFS) and overall survival (OS) between the high-prediction score group and low-prediction score group of RF model. The data of all the patients (n = 44) were used and a *p* value was calculated by log-rank test. PFS was defined as the time from the date of CRT initiation to the date of first radiologic confirmation of tumor progression or death from any cause. OS was defined as the time from the date of CRT initiation to the date of death due to any cause. The follow-up endpoint was set at February 24, 2022. To find possible factors associated with PFS, we used Cox proportional hazards model for univariate and multivariate analyses.

### Statistical analysis

The CR rate of CRT for esophageal squamous carcinoma patients was expected to be 29.6%, according to a previous study^25^. Assuming that the AUC value of our radiomics algorithm for CRT response is 0.9, the sample size (validation cohort) was calculated to be 17, with 80% power and 5% significance level, as determined using Medcalc statistical software. In general, the ratio of the validation cohort and training cohort sample sizes should be reportedly 4:6^15^. Therefore, we set the validation and training cohort sample sizes as 17 and 27, totaling 44 patients.

Statistical differences were analyzed using χ^2^, Fisher exact test or Student t-test. All statistical analyses were performed using R software version 4.0.3, Medcalc statistical software (v.12.7.7., Medcalc Software bvba, Ostend, Belgium), GraphPad Prism version 9.0 (GraphPad Software, San Diego, CA), and JMP software (10.0.2., SAS Institute, Cary, NC). Pearson’s correlation coefficient (r) was used to evaluate the linear relationship between 2 variables. For time-to-event analysis, survival estimates were calculated using Kaplan–Meier analysis, and groups were compared by log-rank test. ROC curves were established to discriminate between CRT responders and non-responders, and the Youden’s index was used to determine the optimal cutoff thresholds for prediction score to predict the CRT response. The prediction score was calculated using the RF model, as described in “Supplementary methods”. According to this formula, a higher score is more likely to show a better response, whereas a lower score is more likely to show a poorer response. The AUCs were compared using DeLong’s test. All *p* values were 2-sided, and those less than 0.05 were considered statistically significant.

## Results

### Patient characteristics

The clinical characteristics of the patients are shown in Table 1. A total of 44 patients were enrolled in this study, including 27 in the training cohort and 17 in the validation cohort. The mean ages were 73.4 years (range 56–96 years) and 68.6 years (range 47–88 years), respectively. A majority of the patients were males; 92.6% and 76.5% respectively. Most patients were T3/4; 85.1% and 70.6%, respectively. The clinical stage was mostly IV that did not have metastatic lesions (M0), namely locally advanced lesions, in both groups. There were 6 (22.2%) CRT responders in the training cohort and 6 (35.3%) in the validation cohort. No significant difference was observed in any of the factors between the 2 groups.

**Table 1:** Patient characteristics.

Characteristic	Training cohort (n = 27)	Validation cohort (n = 17)	*p* Value
Age			0.851
Median	73.4 (56–96)	68.6 (47–88)	
Sex			0.186
Male	25 (92.6%)	13 (76.5%)	
Female	2 (7.4%)	4 (23.5%)	
T stage			0.609
1	2 (7.4%)	2 (11.8%)	
2	2 (7.4%)	3 (17.6%)	
3	12 (44.4%)	5 (29.4%)	
4	11 (40.7%)	8 (41.2%)	
N stage			0.154
0	3 (11.1%)	7 (41.2%)	
1	7 (25.9%)	4 (23.5%)	
2	7 (25.9%)	4 (23.5%)	
3	4 (14.8%)	1 (5.9%)	
4	6 (22.2%)	1 (5.9%)	
M stage			0.716
0	20 (74.1%)	14 (82.4%)	
1*	7 (25.9%)	3 (17.6%)	
Clinical stage			0.187
I	1 (3.7%)	2 (11.8%)	
II	2 (7.4%)	4 (23.5%)	
III	3 (11.1%)	3 (17.6%)	
IV	21 (77.8%)	8 (47.1%)	
Chemotherapy regimen			0.218
NF or FP	17 (63.0%)	7 (41.2%)	
DNF or DFP	10 (37.0%)	10 (58.8%)	
Response			0.489
Responders	6 (22.2%)	6 (35.3%)	
Non-responders	21 (77.8%)	11 (64.7%)	

*NF* nedaplatin/5-FU; *FP* cisplatin/5-FU; *DNF* docetaxel/nedaplatin/5-FU; *DFP* docetaxel/cisplatin/5-FU.

*Patients with distant metastasis stage underwent CRT for local palliative therapy.

### Radiomics features associated with CRT responders

To select the optimal predictive radiomics features associated with the tumor response to CRT, we calculated AUCs for each of the 476 features in the training cohort of ESCC patients. We selected 110 radiomics features with AUCs more than 0.7. We then calculated correlation coefficients among those features, and grouped features with a correlation coefficient (r ≥ 0.7) into 12 groups. The 12 groups and their constituent features are shown in Supplementary Table  S1. The feature with the highest AUC in each of the 12 groups was selected as a CRT susceptibility predictor for ESCC; LLLEnergy, HHLVariance, HLHKurtosis (histogram-based features) and LHLRP, HHHLZE, HHLZP, LHLGHomogeneity2, HHLGContrast, ROIGCorrelation, HLLLRE, ROISRE, and HLHLRE (texture-based features). None of the 12 features were included in the “shape and size-based” features, suggesting that the influence of errors on tumor region delineation should be relatively small. All AUC values of the 12 radiomics features are shown in Supplementary Table S2.

### Radiomics models for CRT response

**Figure 2:**
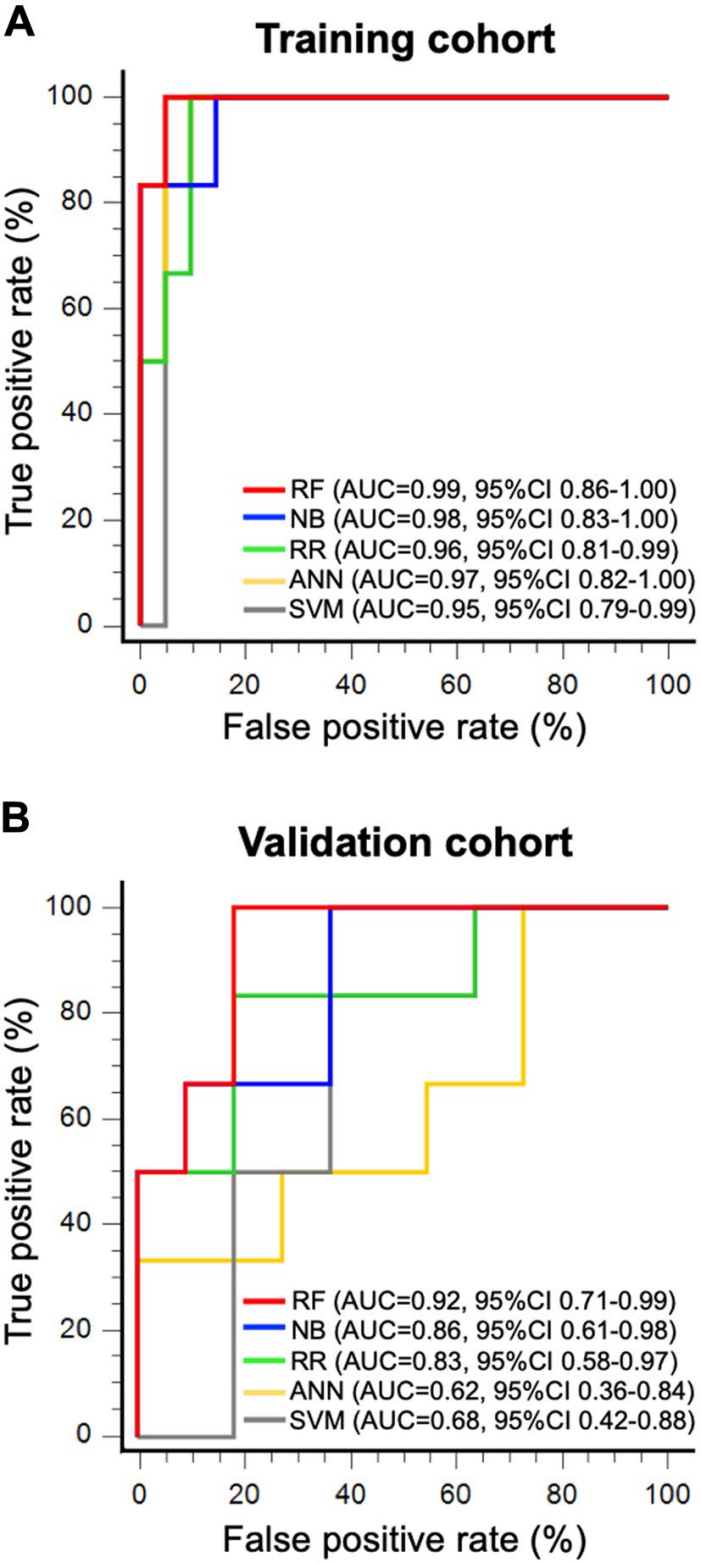
ROC curves plotted by prediction models for each machine learning algorithm. The diagnostic abilities of 5 machine learning models— the RF, NB, RR, ANN, SVM models—were evaluated using ROC curves in the training (**A**) and validation cohorts (**B**). A. Among the 5 machine learning models, the RF model exhibited the highest AUC (0.99 [95%CI 0.86–1.00]) despite showing no significance among the 5 models. B. The RF model showed the highest AUC (0.92 [95%CI 0.71–0.99]), which was significantly higher compared with ANN and SVM by DeLong’s test (*p* < 0.05). The NB and RR did not show any significant difference compared with any of the 5 models.

We used 5 machine learning models (RF, NB, RR, ANN, SVM) to construct radiomics models based on the 12 features. The results for the ROC curves of the CRT response in the training cohort is shown in Fig. 2A. The RF model achieved the highest AUC 0.99 although no significant difference in AUC value was observed among the 5 models. All the radiomics prediction score for the RF model in the training cohort are shown in Supplementary Table S3 (max: 0.449, min: 0.113). Based on the prediction score data in the RF model, the optimal cutoff value was set at 0.19 based on the Youden index; a prediction score ≥ 0.19 represents effectiveness of CRT, and a prediction score < 0.19 represents ineffectiveness of CRT. The accuracy, sensitivity, and specificity of the RF model in the training cohort were 96.3%, 100.0%, and 95.2%, respectively. The other models were also able to show relatively high diagnostic rates: i.e., an AUC of 0.98 for the NB model, 0.96 for the RR model, 0.97 for the ANN model, and 0.95 for the SVM model. Using these 5 machine learning models, we then evaluated ROC curves in the validation cohort (Fig. 2B). Among the 5 models, only the RF model showed significantly higher AUC compared with the ANN and SVM models by DeLong’s test (*p* = 0.01, RF vs SVM; *p* = 0.033, RF vs ANN), although NB and RR did not show any significant difference. Thus, the RF model showed the highest performance (AUC 0.92; accuracy, 82.4%; sensitivity, 83.3%; specificity, 90.0%) for the validation cohort. All the prediction scores of RF model in the validation cohort are shown in Supplementary Table S4. The NB and RR models also showed high AUC values of more than 0.8.

### Survival analysis

**Figure 3:**
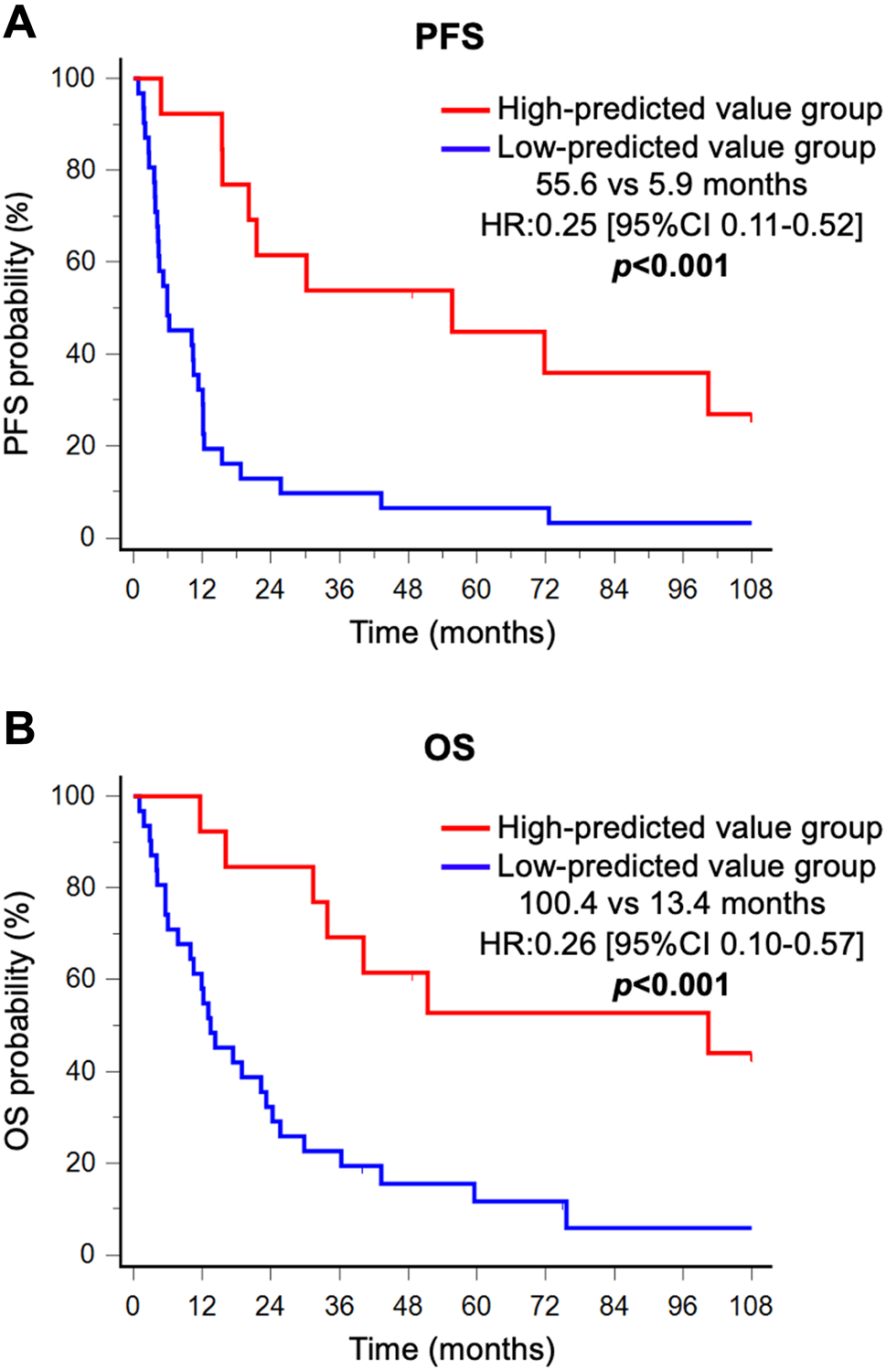
Kaplan–Meier analysis of PFS and OS comparing high- and low-prediction score groups of ESCCs in the RF model. All patients (n = 44) were analyzed using the RF model, categorized as high- or low- prediction score groups, and Kaplan-Meyer curves were drawn. A. Kaplan–Meier curves of PFS. The median PFS in the high-prediction score group was significantly longer than that in the low-prediction score group (55.6 vs 5.9 months; HR:0.25 [95%CI 0.11–0.52]; *p* < 0.001). B. Kaplan–Meier curves of OS. The median OS in the high-prediction score group was significantly longer than that in the low-prediction score group (100.4 vs 13.4 months; HR:0.26 [95%CI 0.10–0.57]; *p* < 0.001).

Since the RF model had the highest prediction performance in the validation phase, we performed survival analyses comparing the high-prediction score group and low-prediction score group in the RF model. In all patients, the PFS in the high-prediction score group was significantly longer than that in the low-prediction score group (55.6 vs 5.9 months; HR:0.25 [95%CI 0.11–0.52]; *p* < 0.001) (Fig. 3A). Similarly, the OS in the high-prediction score group was significantly longer than that in the low-prediction score group (100.4 vs 13.4 months; HR:0.26 [95%CI 0.10–0.57]; *p* < 0.001) (Fig. 3B). Univariate and multivariate Cox regression analysis associated with PFS and OS are shown in Tables 2 and 3. The T stage, lymph node metastasis and radiomics prediction score were significantly associated with both PFS and OS in the univariable analysis. Furthermore, multivariate analysis revealed significant differences in lymph node metastasis (HR:0.41 [95%CI 0.19–0.83]; *p* = 0.013) and radiomics prediction score (HR:0.35 [95%CI 0.14–0.77]; *p* = 0.009) in Table 2, and the T stage (HR:0.26 [95%CI 0.06–0.79]; *p* = 0.014), lymph node metastasis (HR:0.34 [95%CI 0.15–0.70]; *p* = 0.003) and radiomics prediction score (HR:0.44 [95%CI 0.17–0.98]; *p* = 0.056) in Table 3. Similar results were obtained in Kaplan–Meier analysis, and univariate and multivariate analyses in the validation cohort (Supplementary Fig. S2, Tables S5 and S6). Thus, the radiomics prediction score was shown to be an important prognostic factor for ESCC patients treated with CRT.

**Table 2:** Univariate and multivariate analyses of possible factors associated with PFS.

Variable	Univariate analysis			Multivariate analysis		
	HR	95% CI	*p* value	HR	95% CI	*p* value
Age (< Median vs > Median)	0.84	0.44–1.59	0.597			
Gender (Female vs Male)	0.56	0.17–1.42	0.245			
T stage (1–2 vs 3–4)	0.32	0.12–0.73	**0.006**	0.8	0.14–0.77	0.671
Lymph node metastasis (N 0–1 vs 2–4)	0.29	0.14–0.57	** < 0.001**	0.41	0.19–0.83	**0.013**
Serum SCC (≦1.5 vs > 1.5)	0.78	0.41–1.50	0.47			
Tumor location (Mt-Lt vs Ut-Mt)	0.88	0.47–1.67	0.703			
Radiomics prediction score (High vs Low)	0.25	0.11–0.52	** < 0.001**	0.35	0.14–0.77	**0.009**

*PFS* progression free survival; *HR* hazard ratio; *SCC* squamous cell carcinoma.

Significant values in Bold.

**Table 3:** Univariate and multivariate analyses of possible factors associated with OS.

Variable	Univariate analysis			Multivariate analysis		
	HR	95% CI	*p* Value	HR	95% CI	*p* Value
Age (< Median vs > Median)	0.77	0.39–1.51	0.441			
Gender (Female vs Male)	0.42	0.10–1.18	0.108			
T stage (1–2 vs 3–4)	0.16	0.02–0.42	** < 0.001**	0.26	0.06–0.79	**0.014**
Lymph node metastasis (N 0–1 vs 2–4)	0.24	0.11–0.50	** < 0.001**	0.34	0.15–0.70	**0.003**
Serum SCC (≦1.5 vs > 1.5)	0.71	0.36–1.41	0.326			
Tumor location (Mt-Lt vs Ut-Mt)	0.74	0.38–1.46	0.386			
Radiomics prediction score (High vs Low)	0.26	0.10–0.57	** < 0.001**	0.44	0.17–0.98	**0.046**

Significant values in Bold.

## Discussion

In this study, we performed a comprehensive CT-based radiomics analysis to identify candidate features for CRT response from 27 ESCC patients in a training cohort and subsequently identified 12 radiomics features for the CRT response. In addition, we developed a radiomics prediction model for the CRT response with 5 commonly used machine learning algorithms. Thus, we were able to validate high diagnostic performance of the model using another independent CRT cohort of 17 ESCC patients. Furthermore, we expanded survival evaluation and showed a prognostic ability to predict PFS as well as OS. This is the first study proposing a CT-based radiomics model associated with high initial response as well as long-term response after CRT in ESCC patients. Notably, we showed that the radiomics prediction score had superior survival predictability compared with serum SCC-Ag, the most commonly used conventional clinical serological marker for ESCC.

In previous studies, the CRT response was evaluated only a few months after treatment, and radiomics features were analyzed based on such short-term responses because most patients in these studies underwent surgical resection^19,21^. However, in the present study, we defined a CRT responder as `CR of primary lesion in the radiation field maintained for more than 1 year’. Consequently, our model could successfully predict the long-term response after CRT (the median PFS time, 55.6 months). Furthermore, though our study included a variety of patients from early stage to palliative stage and with both resectable and unresectable cancers, most previous studies analyzed only patients who underwent neoadjuvant CRT; ie, resectable patients. Owing to our systematic and comprehensive biomarker approach using the medical data of a total of 44 patients, our radiomics model provided a greater predictability and higher diagnostic accuracy (AUC: 0.92, *p* < 0.001) in comparison with these previous studies^26–30^. Furthermore, the greatest strength of our study is that our radiomics model could predict not only the response to CRT but also the prognosis of ESCC patients who received CRT.

Among the 5 machine learnings (RF, NB, RR, ANN, SVM) used in this study, all the models were predictive with high accuracy rates, especially the RF, NB, and RR models. Our data clearly suggest that the 12 selected features can appropriately predict the CRT response. In particular, the RF model showed the best performance compared with the other models. The RF algorithm uses a number of decision trees and predicts more accurately by averaging the data in case of regression and voting them in case of classification^31^. The RF algorithm can also be used with a wide range of sample sizes including small sample sizes. The characteristics of the RF algorithm may be suitable for the analysis of our data from a relatively small sample size consisting of a wide range of stages (Stage I-IV).

A limitation of this study is that the sample size was comparatively small, and that it was a single-institution retrospective analysis, although radiomics studies with small sample sizes at single institution, similar to our study, have been reported^28,32,33^. Therefore, large multicenter and prospective cohort studies are needed to optimize the generality, robustness, and clinical usefulness of our model. Another limitation is that inter-observer consistency was not evaluated in this study. Intraclass correlation coefficient analysis for this model should be performed in the future.

In conclusion, we used a comprehensive biomarker discovery process with 2 independent clinical cohorts to develop and validate a novel CT-based radiomics model for the prediction of the response to CRT as well as the prognosis of ESCC patients after CRT. Our radiomics model of RF using 12 radiomics features, which is clinically useful, cost-free, and non-invasive, may have the potential to contribute to more effective treatment strategies as a promising and personalized decision-making tool to decrease ESCC mortality.

### Supplementary Information

Supplementary Information.

## Data Availability

The data underlying this article are available on reasonable request to the corresponding author.

## References

[1] Pennathur A, Gibson MK, Jobe BA, Luketich JD (2013). Oesophageal carcinoma. Lancet.

[2] Abnet CC, Arnold M, Wei WQ (2018). Epidemiology of esophageal squamous cell carcinoma. Gastroenterology..

[3] Tanaka Y (2015). Discovery of a good responder subtype of esophageal squamous cell carcinoma with cytotoxic T-lymphocyte signatures activated by chemoradiotherapy. PLoS One..

[4] Lu HW, Chen CC, Chen HH, Yeh HL (2020). The clinical outcomes of elderly esophageal cancer patients who received definitive chemoradiotherapy. J. Chin. Med. Assoc..

[5] Kanamori K (2023). Multimodal therapy for esophageal squamous cell carcinoma according to TNM staging in Japan—A narrative review of clinical trials conducted by Japan Clinical Oncology Group. Annals Esophagus..

[6] Koyanagi K (2020). Progress in multimodal treatment for advanced esophageal squamous cell carcinoma: Results of multi-institutional trials conducted in Japan. Cancers.

[7] Suo D (2020). NRIP3 upregulation confers resistance to chemoradiotherapy in ESCC via RTF2 removal by accelerating ubiquitination and degradation of RTF2. Oncogenesis.

[8] Tixier F (2011). Intratumor heterogeneity characterized by textural features on baseline 18F-FDG PET images predicts response to concomitant radiochemotherapy in esophageal cancer. J. Nucl. Med..

[9] Yip SS (2016). Relationship between the temporal changes in positron-emission-tomography-imaging-based textural features and pathologic response and survival in esophageal cancer patients. Front. Oncol..

[10] Rizzo S (2018). Radiomics: The facts and the challenges of image analysis. Eur. Radiol. Exp..

[11] Lambin P (2012). Radiomics: Extracting more information from medical images using advanced feature analysis. Eur. J. Cancer.

[12] Lambin P (2017). Radiomics: The bridge between medical imaging and personalized medicine. Nat. Rev. Clin. Oncol..

[13] Zhang X, Zhang Y, Zhang G, Qiu X, Tan W, Yin X, Liao L (2022). Deep learning with radiomics for disease diagnosis and treatment: challenges and potential. Front. Oncol..

[14] Arimura H, Soufi M, Kamezawa H, Ninomiya K, Yamada M (2019). Radiomics with artificial intelligence for precision medicine in radiation therapy. J. Radiat. Res..

[15] Pandey AK, Sharma A, Sharma PD, Bal CS, Kumar R (2022). Automated detection of poor-quality scintigraphic images using machine learning. World J. Nucl. Med..

[16] Taghizadeh Kermani A, Ghanbarzadeh R, Joudi Mashhad M, Javadinia SA, Emadi Torghabeh A (2022). predictive value of endoscopic observations and biopsy after neoadjuvant chemoradiotherapy in assessing the pathologic complete response of patients with esophageal squamous cell carcinoma. Front. Oncol..

[17] Aerts HJ (2014). Decoding tumour phenotype by noninvasive imaging using a quantitative radiomics approach. Nat. Commun..

[18] Haga A (2018). Classification of early stage non-small cell lung cancers on computed tomographic images into histological types using radiomic features: interobserver delineation variability analysis. Radiol. Phys. Technol..

[19] Hu Y (2020). Assessment of intratumoral and peritumoral computed tomography radiomics for predicting pathological complete response to neoadjuvant chemoradiation in patients with esophageal squamous cell carcinoma. JAMA Netw. Open..

[20] Wu J (2019). Radiomics analysis of iodine-based material decomposition images with dual-energy computed tomography imaging for preoperatively predicting microsatellite instability status in colorectal cancer. Front. Oncol..

[21] Hu Y (2021). Computed tomography-based deep-learning prediction of neoadjuvant chemoradiotherapy treatment response in esophageal squamous cell carcinoma. Radiother. Oncol..

[22] Wu W (2016). Exploratory study to identify radiomics classifiers for lung cancer histology. Front. Oncol..

[23] Jeswal, S. K. & Chakraverty, S. Fuzzy eigenvalue problems of structural dynamics using ANN *New Paradigms in Computational Modeling and Its Applications*, 145–161 (2021).

[24] Huang S (2018). Applications of support vector machine (SVM) learning in cancer genomics. Cancer Genomics Proteomics.

[25] Liu X (2017). The lymphocyte-monocyte ratio predicts tumor response and survival in patients with locally advanced esophageal cancer who received definitive chemoradiotherapy. OncoTargets Ther..

[26] Jin X (2019). Prediction of response after chemoradiation for esophageal cancer using a combination of dosimetry and CT radiomics. Eur. Radiol..

[27] Yang Z (2019). CT-based radiomic signatures for prediction of pathologic complete response in esophageal squamous cell carcinoma after neoadjuvant chemoradiotherapy. J. Radiat. Res..

[28] Hou Z (2017). Radiomic analysis in contrast-enhanced CT: Predict treatment response to chemoradiotherapy in esophageal carcinoma. Oncotarget.

[29] Qiu Q (2020). Development and validation of a radiomics nomogram model for predicting postoperative recurrence in patients with esophageal squamous cell cancer who achieved pCR after neoadjuvant chemoradiotherapy followed by surgery. Front. Oncol..

[30] Xie C (2019). Sub-region based radiomics analysis for survival prediction in oesophageal tumours treated by definitive concurrent chemoradiotherapy. EBioMedicine.

[31] Xu C, Wang J, Zheng T, Cao Y, Ye F (2022). Prediction of prognosis and survival of patients with gastric cancer by a weighted improved random forest model: an application of machine learning in medicine. Arch. Med. Sci..

[32] Buizza G (2021). Radiomics and dosiomics for predicting local control after carbon-ion radiotherapy in skull-base chordoma. Cancers.

[33] Yang J, Guo X, Ou X, Zhang W, Ma X (2019). Discrimination of pancreatic serous cystadenomas from mucinous cystadenomas with CT textural features: based on machine learning. Front. Oncol..

