# Author Correction: A novel CT-based radiomics model for predicting response and prognosis of chemoradiotherapy in esophageal squamous cell carcinoma

**DOI:** 10.1038/s41598-024-54210-w

**Published:** 2024-02-13

**Authors:** Akinari Kasai, Jinsei Miyoshi, Yasushi Sato, Koichi Okamoto, Hiroshi Miyamoto, Takashi Kawanaka, Chisato Tonoiso, Masafumi Harada, Masakazu Goto, Takahiro Yoshida, Akihiro Haga, Tetsuji Takayama

**Affiliations:** 1 Department of Gastroenterology and Oncology, Tokushima University Graduate School of Biomedical Sciences, 3-18-15 Kuramoto-cho, Tokushima, 770-8503 Japan; 2 Department of Gastroenterology, Kawashima Hospital, Tokushima, Japan; 3 Department of Radiology, Tokushima University Graduate School of Biomedical Sciences, Tokushima, Japan; 4 Department of Thoracic, Endocrine Surgery and Oncology, Tokushima University Graduate School of Biomedical Sciences, Tokushima, Japan; 5 Yoshida Clinic, Tokushima, Japan; 6 Department of Medical Image Informatics, Tokushima University Graduate School of Biomedical Sciences, Tokushima, Japan

Correction to: *Scientific Reports* 10.1038/s41598-024-52418-4, published online 23 January 2024

The original version of this Article contained an error in the Methods section.

Under the subheading, ‘CT imaging protocol’,

“The CT scanning parameters included a tube voltage of 120 kV, tube current auto, pixel size 0.976 × 0.976  0mm^2^, and slice thickness 2.5 mm.”

now reads:

“The CT scanning parameters included a tube voltage of 120 kV, tube current auto, pixel size 0.976 × 0.976  mm^2^, and slice thickness 2.5 mm.”

The original Article has been corrected.

