# Supplementary material for: A novel CT-based radiomics model for predicting response and prognosis of chemoradiotherapy in esophageal squamous cell carcinoma
Source: Sci Rep. 2024 Jan 23;14:2039. doi: 10.1038/s41598-024-52418-4 (PMC10806175; doi:10.1038/s41598-024-52418-4)
Supplement: Supplementary file 1 — Supplementary Information. [file 41598_2024_52418_MOESM1_ESM.pdf]

## Supplementary information

**A Novel CT-Based Radiomics Model for Predicting Response and Prognosis of Chemoradiotherapy in Esophageal Squamous Cell Carcinoma**

Akinari Kasai[1], Jinsei Miyoshi[1,2], Yasushi Sato[1], Koichi Okamoto[1], Hiroshi Miyamoto[1], Takashi Kawanaka[3], Chisato Tonoiso[3], Masafumi Harada[3], Masakazu Goto[4], Takahiro Yoshida[4,5], Akihiro Haga[6], Tetsuji Takayama[1]*

1) Department of Gastroenterology and Oncology, Tokushima University Graduate School of Biomedical Sciences, 3-18-15 Kuramoto-cho, Tokushima 770-8503, Japan
2) Department of Gastroenterology, Kawashima Hospital, Tokushima, Japan
3) Department of Radiology, Tokushima University Graduate School of Biomedical Sciences, Tokushima, Japan
4) Department of Thoracic, Endocrine Surgery and Oncology, Tokushima University Graduate School of Biomedical Sciences, Tokushima, Japan
5) Yoshida Clinic, Tokushima, Japan
6) Department of Medical Image Informatics, Tokushima University Graduate School of Biomedical Sciences, Tokushima, Japan

## Supplementary methods

## Prediction score calculation

The prediction score was calculated using the following formula based on the RF model algorithm, as described previously (1):

$$C_\alpha(T) = \sum_{m=1}^{|T|} N_m Q_m(T) + \alpha|T|$$

We drew a bootstrap sample $Z^b$ from the training or validation cohort. We selected m variables at random from the total variables and picked the best variable/split-point among the $m$, then split the node into 2 daughter nodes, repeated the following steps for each terminal node of the tree until the minimum node size $n_{min}$ grew to a random forest tree $T_b$. We grew a tree by changing the sample set B times and outputted the ensemble of trees $\{T_b\}_1^B$ . The GainRatio is used for Q, the maximum number of split points in the tree is 5, and the number of repeating is B=50. [1]

## Reference


1. Goldstein, B. A., Polley, E. C. & Briggs, F. B. Random forests for genetic association studies. *Stat Appl Genet Mol Biol*. **10**, 32 (2011).

**Supplementary Figure legends**

**Supplementary Figure S1.**

The process of radiomics features extraction. The CT image set is decomposed into 8 wavelet-transformed sets, and 52 features (texture-based and histogram-based features) are extracted from each set, as well as from the original image set. We analyzed texture-based features extracted from CT images using gray level run length matrix (GLRLM), gray level size zone matrix (GLSZM), neighboring gray-tone difference matrix (NGTDM), and gray level cooccurrence matrix (GLCM) techniques. Eight shape and size-based features are independently extracted from the images. In total, 476 features are extracted from each CT image set.

**Supplementary Figure S2.**

Kaplan-Meyer analysis of high- and low-prediction score groups of ESCCs in the RF model. Validation cohort patients were analyzed using the RF model, categorized as high- or low-prediction score groups, and Kaplan-Meyer curves were drawn. (a) Kaplan-Meier curves of PFS. The median PFS in the high-prediction score group was significantly longer than that in the low-prediction score group (100.4 vs 4.3 months; HR:0.20 [95%CI 0.04-0.71]; *p*=0.012). (b) Kaplan-Meier curves of OS. The median OS was not reached in the high-prediction score group, and the median OS in the high-prediction score group was significantly longer than 10.5 months in the low-prediction score group (HR:0.22 [95%CI 0.03-0.94]; *p*=0.041).

**Supplementary Figure S1**

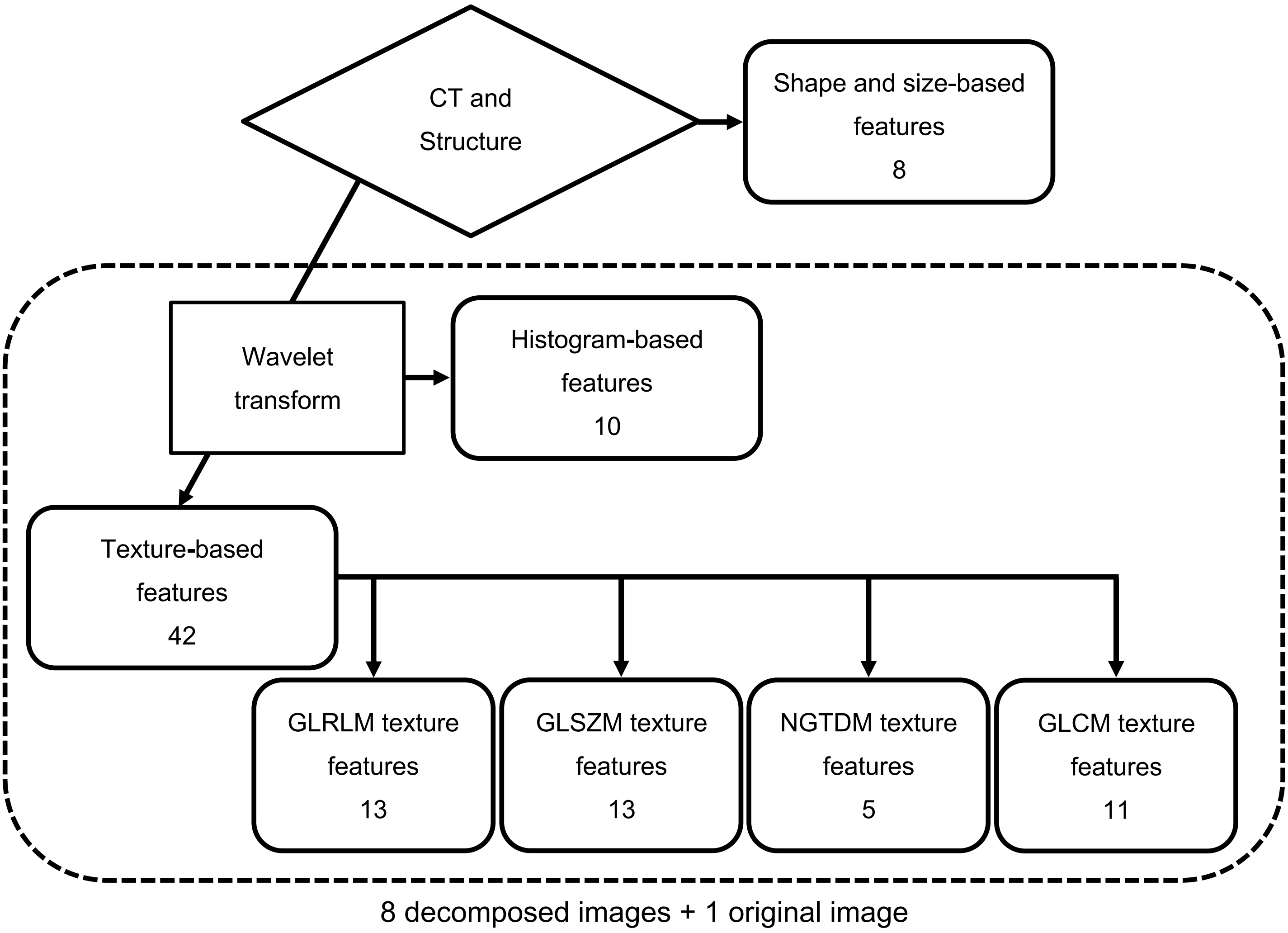

**Supplementary Figure S2**

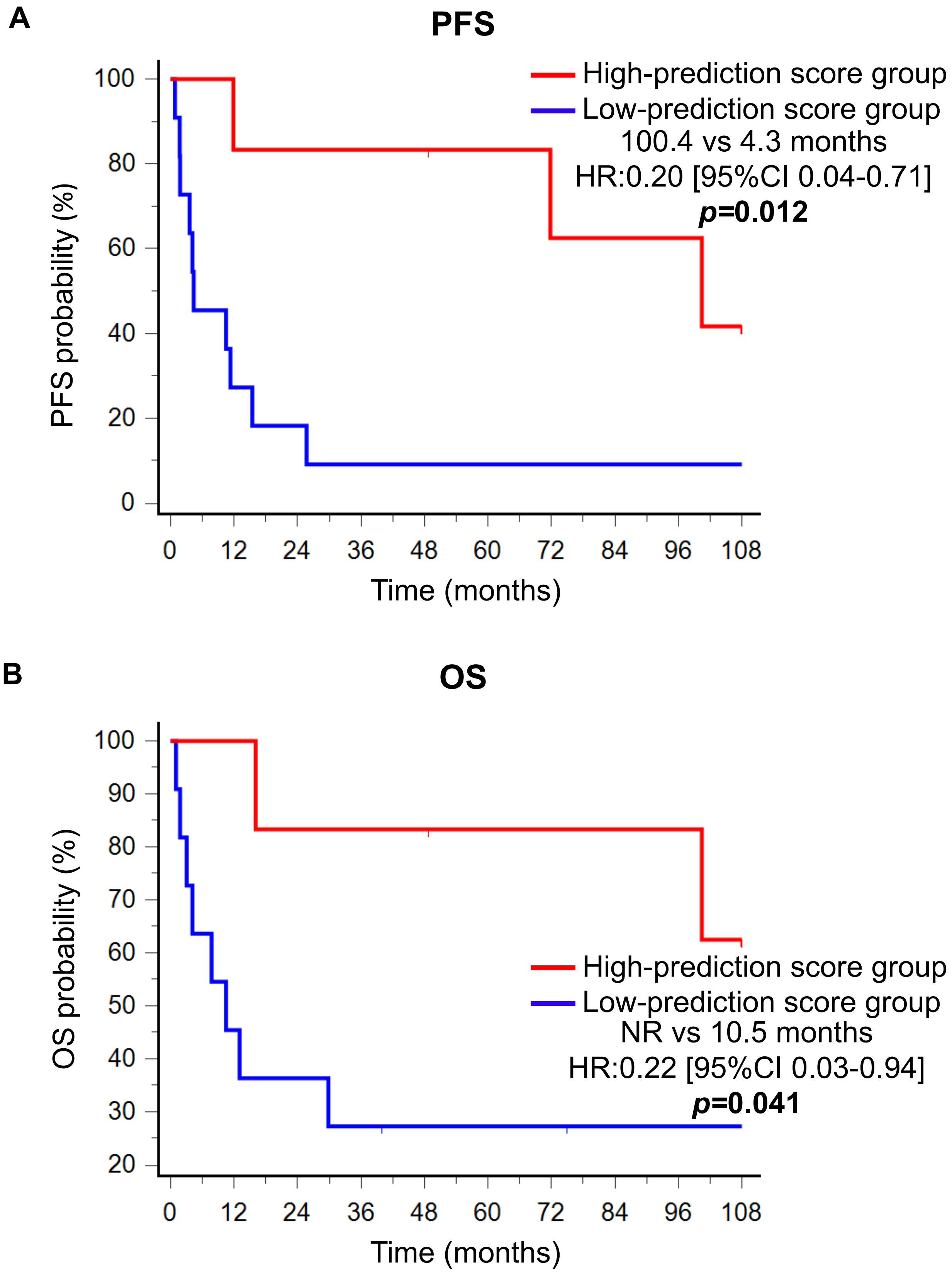

**Supplementary Table S1.** Selected radiomic features associated with responders.

| Selected 110 features | Highest AUC features in each 12 groups | Feature types |
|---|---|---|
| LHLRP LHLSRE LHLRLN LHLLRE LHHLRE LHHRP HHLLRE LHHSRE LHHRLN HHLSRE HHLRLN HHLRP HHHRLN HHHLRE | LHLRP | GLRLM |
| HHHLZE HHLLZE HHLLZHGE LHLLZHGE LHHLZHGE HHHLZLGE HHHLZHGE LHLLZE HHLBusyness ROILZHGE HLHLZHGE HHLLZLGE LLHLZHGE LHHLZE LLLLZHGE HLLLZHGE | HHHLZE | GLSZM |
| HHHZP HHLZP LLHMedian HHLGLV ROICoarseness HLLCoarseness LHLzGLV LHHzGLV LLLCoarseness LHHZP LHLCoarseness HLHzGLV HHLGVariance LHLZP HHLzGLV HHHzGLV LHHCoarseness LHLGEnergy LLHCoarseness HHLGLN HLHCoarseness HHLContrast HHLStrength LHHContrast HHLCoarseness LHLContrast HHHGVariance HHHUniformity HHHCoarseness LLHRLV HLLzGLV HLHMedian LHLGCorrelation LHHGEnergy HLHRLV HHHStrength LHHRLV HLLRLV HHHEntropy LHLRLV | HHHZP | GLSZM |
| LLLEnergy LHLEnergy HLLEnergy Volume ROIEnergy LLHEnergy LHHEnergy HLHEnergy HHLEnergy HHHEnergy Max3Ddiameter Compactness1 SurfaceArea | LLLEnergy | Histogram |
| LHLGHomogeneity2 LHLGHomogeneity1 HHLGHomogeneity2 HHLGEntropy HHLGHomogeneity1 HHLGEnergy LHHGHomogeneity1 LHHGHomogeneity2 HHLLRLGE | LHLGHomogeneity2 | GLCM |
| HHLGContrast HHHGContrast | HHLGContrast | GLCM |
| HHLVariance HHHVariance HLHVariance LHLVariance | HHLVariance | Histogram |
| HLHKurtosis HHHKurtosis | HLHKurtosis | Histogram |
| ROIGCorrelation LLLGCorrelation | ROIGCorrelation | GLCM |
| HLLLRE | HLLLRE | GLRLM |
| ROISRE ROILRE ROIRLN | ROISRE | GLRLM |
| HLHLRE | HLHLRE | GLRLM |

**Supplementary Table S2.** AUC analysis data of the 12 radiomics features.

| Patient No. | CRT response | LHLRP | HHHLZE | HHHZP | LLLEnergy | LHLGHomo geneity2 | HHLG Contrast | HHL Contrast | HLH Kurtosis | ROIG Correlation | HLLLRE | ROISRE | HLHLRE |
|---|---|---|---|---|---|---|---|---|---|---|---|---|---|
| 1 | Non-responder | 0.811007 | 66679.4138 | 0.031522 | 4.84E+10 | 0.459677 | 1.214478 | 157.680966 | 20.047932 | 0.357968 | 2.436486 | 0.884449 | 3.450812 |
| 2 | Non-responder | 0.775034 | 67106.0820 | 0.023726 | 3.37E+10 | 0.533440 | 1.183136 | 48.7504870 | 414.77330 | 0.204916 | 5.906001 | 0.758087 | 5.205147 |
| 3 | Non-responder | 0.815462 | 63630.0690 | 0.032880 | 4.33E+10 | 0.437211 | 1.095923 | 286.854829 | 10.756778 | 0.724624 | 2.720220 | 0.883970 | 5.380646 |
| 4 | Non-responder | 0.664523 | 239076.509 | 0.017998 | 1.19E+11 | 0.592646 | 1.109298 | 104.908168 | 30.621356 | 0.644514 | 3.256775 | 0.760694 | 10.984914 |
| 5 | Non-responder | 0.597798 | 237566.663 | 0.024956 | 8.73E+10 | 0.682245 | 0.380078 | 74.3773200 | 104.61192 | 0.459181 | 4.581314 | 0.725012 | 4.091106 |
| 6 | Non-responder | 0.810774 | 65906.3333 | 0.021494 | 3.46E+10 | 0.435623 | 1.703535 | 217.745056 | 14.029491 | 0.696498 | 2.142096 | 0.922028 | 2.964659 |
| 7 | Non-responder | 0.764140 | 23114.0389 | 0.026540 | 5.64E+11 | 0.516403 | 0.898892 | 179.454178 | 19.048502 | 0.624840 | 2.746734 | 0.848756 | 2.904978 |
| 8 | Non-responder | 0.834441 | 51350.4098 | 0.052136 | 7.97E+10 | 0.405740 | 2.479750 | 243.566678 | 15.173564 | 0.502946 | 2.017851 | 0.846137 | 2.401004 |
| 9 | Responder | 0.825017 | 43982.2833 | 0.050761 | 6.05E+10 | 0.431255 | 1.653293 | 224.760839 | 10.757543 | 0.593596 | 2.532296 | 0.853384 | 3.443339 |
| 10 | Responder | 0.835642 | 22062.0089 | 0.050970 | 2.86E+10 | 0.449841 | 1.226140 | 708.698888 | 12.062720 | 0.425756 | 2.531662 | 0.868513 | 3.757263 |
| 11 | Non-responder | 0.808398 | 111260.970 | 0.036874 | 1.17E+11 | 0.449614 | 1.510976 | 51.1667280 | 163.11940 | 0.530524 | 2.496468 | 0.777444 | 4.749182 |
| 12 | Non-responder | 0.705548 | 497277.611 | 0.031651 | 3.63E+11 | 0.583430 | 0.805112 | 111.480510 | 26.738300 | 0.531267 | 3.529906 | 0.785144 | 5.094176 |
| 13 | Responder | 0.811700 | 55481.3615 | 0.045907 | 4.65E+10 | 0.457767 | 2.236047 | 97.3768740 | 91.074049 | 0.335686 | 3.202451 | 0.846928 | 4.258215 |
| 14 | Non-responder | 0.774921 | 168787.401 | 0.046879 | 1.61E+11 | 0.510742 | 0.773738 | 50.1312960 | 157.11193 | 0.500176 | 3.924734 | 0.827591 | 3.932343 |
| 15 | Non-responder | 0.791644 | 292143.044 | 0.028165 | 2.27E+11 | 0.472537 | 1.382582 | 26.3840810 | 553.72341 | 0.295769 | 3.544938 | 0.699059 | 4.072064 |
| 16 | Non-responder | 0.807372 | 28499.6989 | 0.045015 | 2.37E+10 | 0.447793 | 1.527224 | 183.097728 | 129.74530 | 0.739737 | 2.289269 | 0.867582 | 2.386470 |
| 17 | Responder | 0.866560 | 17776.6612 | 0.049959 | 3.11E+10 | 0.370786 | 2.516368 | 653.524849 | 4.0048610 | 0.267963 | 2.203315 | 0.872940 | 3.107109 |
| 18 | Non-responder | 0.790412 | 118449.527 | 0.026829 | 8.69E+10 | 0.510838 | 0.645034 | 373.494233 | 28.549085 | 0.525896 | 3.416551 | 0.837696 | 5.194502 |
| 19 | Non-responder | 0.806354 | 202726.763 | 0.035508 | 2.01E+11 | 0.454060 | 1.522172 | 77.0008930 | 109.17868 | 0.623487 | 3.316903 | 0.797372 | 6.399303 |
| 20 | Non-responder | 0.766490 | 69181.7590 | 0.058061 | 7.69E+10 | 0.522746 | 1.076556 | 56.7866060 | 195.76206 | 0.588959 | 2.695496 | 0.839248 | 4.035754 |
| 21 | Non-responder | 0.744826 | 161831.647 | 0.024806 | 7.89E+10 | 0.536896 | 0.871097 | 198.071513 | 14.867853 | 0.628417 | 2.442536 | 0.887672 | 4.277500 |
| 22 | Non-responder | 0.808250 | 83531.3440 | 0.033035 | 9.03E+10 | 0.466147 | 1.598329 | 645.187570 | 1.8568740 | 0.471667 | 2.425042 | 0.868009 | 3.232313 |
| 23 | Non-responder | 0.818036 | 24969.7238 | 0.046113 | 2.95E+10 | 0.476361 | 1.431400 | 435.955159 | 19.574760 | 0.401367 | 2.654561 | 0.850187 | 4.163343 |
| 24 | Responder | 0.851522 | 9491.70968 | 0.081507 | 3.01E+10 | 0.403041 | 4.423346 | 125.820642 | 62.646542 | 0.210096 | 2.125035 | 0.819748 | 2.396486 |
| 25 | Non-responder | 0.811732 | 56652.5103 | 0.038257 | 6.67E+10 | 0.461522 | 2.067658 | 52.5139000 | 145.32328 | 0.363421 | 2.248957 | 0.788485 | 2.778597 |
| 26 | Responder | 0.814887 | 46533.3475 | 0.048520 | 7.62E+10 | 0.477346 | 1.302931 | 533.599964 | 9.1102060 | 0.501139 | 2.320010 | 0.886685 | 3.034703 |
| 27 | Non-responder | 0.799562 | 78059.2481 | 0.016364 | 1.58E+12 | 0.414558 | 3.181300 | 87.6320590 | 50.631546 | 0.699512 | 4.447133 | 0.762356 | 4.119252 |
| | AUC | 0.937 | 0.921 | 0.905 | 0.817 | 0.786 | 0.786 | 0.778 | 0.762 | 0.762 | 0.722 | 0.714 | 0.706 |

**Supplementary Table S3.** Radiomics prediction score for RF model in training cohort.

| Patient No. | Response | Radiomics prediction score |
|---|---|---|
| 1 | Non-responder | 0.16380256 |
| 2 | Non-responder | 0.18189779 |
| 3 | Non-responder | 0.18470515 |
| 4 | Non-responder | 0.11303488 |
| 5 | Non-responder | 0.13248942 |
| 6 | Non-responder | 0.17485126 |
| 7 | Non-responder | 0.13435523 |
| 8 | Non-responder | 0.28856775 |
| 9 | Responder | 0.32379065 |
| 10 | Responder | 0.38385097 |
| 11 | Non-responder | 0.11303488 |
| 12 | Non-responder | 0.11303488 |
| 13 | Responder | 0.27074018 |
| 14 | Non-responder | 0.15948942 |
| 15 | Non-responder | 0.14487038 |
| 16 | Non-responder | 0.17138264 |
| 17 | Responder | 0.44865873 |
| 18 | Non-responder | 0.12053488 |
| 19 | Non-responder | 0.11303488 |
| 20 | Non-responder | 0.11541583 |
| 21 | Non-responder | 0.11303488 |
| 22 | Non-responder | 0.14564931 |
| 23 | Non-responder | 0.13533719 |
| 24 | Responder | 0.35564495 |
| 25 | Non-responder | 0.17311172 |
| 26 | Responder | 0.35706165 |
| 27 | Non-responder | 0.14927121 |

**Supplementary Table S4.** Radiomics prediction score for RF model in validation cohort.

| Patient No. | Response | Radiomics prediction score |
|---|---|---|
| 28 | Non-responder | 0.18442124 |
| 29 | Non-responder | 0.14636821 |
| 30 | Non-responder | 0.14963228 |
| 31 | Responder | 0.30205444 |
| 32 | Non-responder | 0.11303488 |
| 33 | Non-responder | 0.14636821 |
| 34 | Responder | 0.19842962 |
| 35 | Non-responder | 0.15756230 |
| 36 | Non-responder | 0.21780003 |
| 37 | Responder | 0.17701684 |
| 38 | Non-responder | 0.11303488 |
| 39 | Responder | 0.36431662 |
| 40 | Responder | 0.35564495 |
| 41 | Non-responder | 0.13248942 |
| 42 | Responder | 0.19775458 |
| 43 | Non-responder | 0.13248942 |
| 44 | Non-responder | 0.15756230 |

**Supplementary Table S5.** Univariate and multivariate analyses of possible factors associated with PFS in validation cohort.

| Variable | Univariate analysis | | | Multivariate analysis | | |
|---|---|---|---|---|---|---|
| | HR | 95% CI | *p* value | HR | 95% CI | *p* value |
| Age (<Median vs >Median) | 0.47 | 0.15-1.48 | 0.209 | | | |
| Gender (Female vs Male) | 0.37 | 0.06-1.41 | 0.165 | | | |
| T stage (1-2 vs 3-4) | 0.32 | 0.07-1.09 | 0.078 | | | |
| Lymph node metastasis (N 0-1 vs 2-4) | 0.13 | 0.03-0.50 | **0.005** | 0.09 | 0.01-0.42 | **0.002** |
| Serum SCC (≦1.5 vs >1.5) | 0.48 | 0.15-1.55 | 0.228 | | | |
| Tumor location (Mt-Lt vs Ut-Mt) | 0.54 | 0.18-1.79 | 0.306 | | | |
| Radiomics prediction score (High vs Low) | 0.20 | 0.04-0.71 | **0.012** | 0.16 | 0.03-0.62 | **0.006** |

PFS, progression free survival; HR, hazard ratio; SCC, squamous cell carcinoma.

**Supplementary Table S6.** Univariate and multivariate analyses of possible factors associated with OS in validation cohort.

| Variable | Univariate analysis | | | Multivariate analysis | | |
|---|---|---|---|---|---|---|
| | HR | 95% CI | *p* value | HR | 95% CI | *p* value |
| Age (<Median vs >Median) | 0.28 | 0.07-0.97 | **0.046** | 0.31 | 0.07-1.33 | 0.115 |
| Gender (Female vs Male) | 0.28 | 0.02-1.50 | 0.155 | | | |
| T stage (1-2 vs 3-4) | 0.14 | 0.01-0.79 | **0.022** | 0.76 | 0.03-8.45 | 0.832 |
| Lymph node metastasis (N 0-1 vs 2-4) | 0.10 | 0.01-0.43 | **0.002** | 0.12 | 0.01-0.72 | **0.018** |
| Serum SCC (≦1.5 vs >1.5) | 0.71 | 0.20-2.80 | 0.603 | | | |
| Tumor location (Mt-Lt vs Ut-Mt) | 0.55 | 0.16-2.17 | 0.372 | | | |
| Radiomics prediction score (High vs Low) | 0.22 | 0.03-0.94 | **0.041** | 0.17 | 0.02-1.05 | 0.057 |

OS, overall survival; HR, hazard ratio; SCC, squamous cell carcinoma.
